# Musculoskeletal conditions may increase the risk of chronic disease: a systematic review and meta-analysis of cohort studies

**DOI:** 10.1186/s12916-018-1151-2

**Published:** 2018-09-25

**Authors:** Amanda Williams, Steven J. Kamper, John H. Wiggers, Kate M. O’Brien, Hopin Lee, Luke Wolfenden, Sze Lin Yoong, Emma Robson, James H. McAuley, Jan Hartvigsen, Christopher M. Williams

**Affiliations:** 1 School of Medicine and Public Health, University of Newcastle, Callaghan, NSW 2308 Australia; 2 Hunter New England Population Health, Locked Bag 10, Wallsend, NSW 2287 Australia; 3 Centre for Pain, Health and Lifestyle, Ourimbah, NSW Australia; 4 School of Public Health, University of Sydney, Lvl 10, King George V Building, Camperdown, NSW 2050 Australia; 5 Neuroscience Research Australia (NeuRA), PO Box 1170, Randwick, NSW 2031 Australia; 6 Nuffield Department of Orthopaedics Rheumatology and Musculoskeletal Sciences, Centre for Statistics in Medicine, University of Oxford, Oxford, UK; 7 Department of Sports Science and Clinical Biomechanics, University of Southern Denmark, Campusvej 55, 5230 Odense M, Denmark; 8 Nordic Institute of Chiropractic and Clinical Biomechanics, Campusvej 55, 5230 Odense M, Denmark

**Keywords:** Osteoarthritis, Back pain, Chronic disease, Meta-analysis

## Abstract

**Background:**

Chronic diseases and musculoskeletal conditions have a significant global burden and frequently co-occur. Musculoskeletal conditions may contribute to the development of chronic disease; however, this has not been systematically synthesised. We aimed to investigate whether the most common musculoskeletal conditions, namely neck or back pain or osteoarthritis of the knee or hip, contribute to the development of chronic disease.

**Methods:**

We searched CINAHL, Embase, Medline, Medline in Process, PsycINFO, Scopus and Web of Science to February 8, 2018, for cohort studies reporting adjusted estimates of the association between baseline musculoskeletal conditions (neck or back pain or osteoarthritis of the knee or hip) and subsequent diagnosis of a chronic disease (cardiovascular disease, cancer, diabetes, chronic respiratory disease or obesity). Two independent reviewers performed data extraction and assessed study quality. Adjusted hazard ratios were pooled using the generic inverse variance method in random effect models, regardless of the type of musculoskeletal condition or chronic disease. PROSPERO: CRD42016039519.

**Results:**

There were 13 cohort studies following 3,086,612 people. In the primary meta-analysis of adjusted estimates, osteoarthritis (*n* = 8 studies) and back pain (*n* = 2) were the exposures and cardiovascular disease (*n* = 8), cancer (*n* = 1) and diabetes (*n* = 1) were the outcomes. Pooled adjusted estimates from these 10 studies showed that people with a musculoskeletal condition have a 17% increase in the rate of developing a chronic disease compared to people without (hazard ratio 1.17, 95% confidence interval 1.13–1.22; I^2^ 52%, total *n* = 2,686,113 people).

**Conclusions:**

This meta-analysis found that musculoskeletal conditions may increase the risk of chronic disease. In particular, osteoarthritis appears to increase the risk of developing cardiovascular disease. Prevention and early treatment of musculoskeletal conditions and targeting associated chronic disease risk factors in people with long standing musculoskeletal conditions may play a role in preventing other chronic diseases. However, a greater understanding about why musculoskeletal conditions may increase the risk of chronic disease is needed.

**Electronic supplementary material:**

The online version of this article (10.1186/s12916-018-1151-2) contains supplementary material, which is available to authorized users.

## Background

Non-communicable chronic diseases are responsible for a significant global burden of disease. Cardiovascular disease, cancer, diabetes and chronic respiratory diseases ranked among the leading causes of global disability-adjusted life years in 2015 [1]. Together, these conditions were responsible for over 31 million of 56 million deaths worldwide in 2012 [2]. Obesity, now also considered a chronic disease [3], also contributes to a high rate of morbidity and all-cause mortality [2].

Another significant source of the global disease burden is from musculoskeletal conditions, specifically neck and back pain as well as osteoarthritis (OA) of the knee and/or hip [1, 4]. Neck and back pain ranks fourth among the leading causes of disability-adjusted life years, and elderly people with neck and back pain or OA die sooner than those without [1, 5, 6]. When considering only years lived with disability (YLDs), neck and back pain, as well as OA, rank 1st and 13th, respectively, among all causes of global YLDs and together accounted for 13.6% of YLDs in 2015 [4].

Evidence shows that chronic diseases and musculoskeletal conditions frequently co-occur [7] and, importantly, people with musculoskeletal conditions are reported to have roughly a two-fold chance of having chronic disease of other body systems such as heart disease, neurological disorders, gastric ulcers and endocrine disorders [8]. Several mechanisms have been proposed to explain these links. Chronic inflammation associated with OA has been hypothesised to increase the risk of cardiovascular disease, diabetes and cancer [6]. Pain and disability from musculoskeletal conditions can also limit participation in physical activity, or may influence other risk factors for chronic diseases, for example, weight gain or poor sleep [6, 8–11]. Similarly, pain management approaches which are widely used for back pain and OA, for example, the use of non-steroidal anti-inflammatory drugs, are known to increase the risk of cardiovascular events and mortality [6]. These hypotheses suggest musculoskeletal pain may play a role in the subsequent development of other chronic diseases.

Nevertheless, despite the suggested link between musculoskeletal conditions and chronic diseases [6, 9, 12, 13], to the best of our knowledge, the available studies have not been systematically synthesised and a direct longitudinal relationship has not been considered. To determine if the most common and burdensome musculoskeletal conditions (neck or back pain or OA of the knee or hip) increased the development of chronic disease (cardiovascular disease, cancer, diabetes, chronic respiratory disease and obesity) we conducted a systematic review and meta-analysis of longitudinal cohort studies reporting adjusted estimates of the association between these musculoskeletal conditions and the development of chronic disease.

## Methods

The systematic review protocol was prospectively registered with Prospero on May 24, 2016 (PROSPERO 2016, CRD42016039519: https://www.crd.york.ac.uk/prospero/display_record.php?RecordID=39519). The systematic review adheres to the Meta-analysis of Observational Studies in Epidemiology (MOOSE) reporting guideline [14].

### Study eligibility

We included longitudinal cohort studies that estimated a direct association between baseline neck or back pain or OA of the knee or hip (i.e. exposure) and subsequent diagnosis of a chronic disease (cardiovascular disease, cancer, diabetes, chronic respiratory disease or obesity) over any follow-up length (i.e. outcome). We did not aim to identify studies of mechanisms or specific causal factors for chronic disease such as treatment provided (e.g. non-steroidal anti-inflammatory drugs) or features of pain (e.g. disabling pain). Studies with mixed populations of musculoskeletal conditions where separate data was provided for the conditions of interest, or where at least 75% of the ‘musculoskeletal conditions’ reported were one of or a combination of neck or back pain or OA of the knee or hip, were included. Studies assessing specific forms of OA other than knee or hip (e.g. hand/wrist, foot, etc.) were excluded. We included any study that assessed ‘osteoarthritis’ broadly (i.e. did not define the type of OA) as we expected knee and hip OA would constitute the majority of participants, as these are the most prevalent forms of OA [15]. We included all neck or back pain and OA, defined as clinical, self-reported and diagnoses with or without imaging. There were no restrictions on the study setting, participant age, length of follow-up, publication type (e.g. abstracts from conference proceedings, dissertations), publication date or language.

### Data sources and search strategy

We searched CINAHL, Embase, Medline, Medline in Process, PsycINFO, Scopus and Web of Science for eligible studies. Databases were searched from inception to February 8, 2018. The search used key terms as subject headings and text words to identify (1) neck or back pain or OA of the knee or hip, and (2) chronic diseases (cardiovascular disease, cancer, diabetes, chronic respiratory disease or obesity) along with terms for chronic disease and morbidity (Additional file 1: Table S1). The search strategy was reviewed and performed by an information specialist. We manually searched the reference lists of included studies to identify further studies. All references were stored in Endnote X7 software.

### Study selection

Before screening, duplicates were removed using the duplicate removal function in Endnote X7 software. After removing duplicates, pairs of review authors independently screened studies for inclusion based on title and abstract (AW, SK, KO, SY, ER, CW). For studies not excluded at this step, each full text retrieved was screened independently by pairs of review authors to determine final inclusion (AW, SK, KO, CW). Consensus was used to resolve any disagreements and a third reviewer was consulted when required (SK, CW). Articles written in a language other than English were screened by a researcher with relevant language skills, these researchers either worked in the same institution as one of the authors or were familiar to an author via collaboration on previous projects.

### Data extraction

Relevant information was extracted from included studies by one author and checked for accuracy and omissions by a second author (AW, KO). Discrepancies were resolved by discussion. The following study characteristic information was extracted into nine categories, as outlined in Additional file 1: Table S2: study source and country, population description, number of patients with a musculoskeletal condition, age, sex, measure of musculoskeletal condition, measure of chronic disease, follow-up time and adjustment for any covariates. All information was extracted directly into the table. Outcome data (i.e. estimates of the association between a musculoskeletal condition and a chronic disease) were extracted into Microsoft Excel 2013 and then those estimates used in meta-analyses were stored in RevMan5 software for analysis [16].

### Risk of bias assessment

Risk of bias was assessed using a modified version of the Quality in Prognosis Studies (QUIPS) tool for assessing studies of prognostic factors [17]. For this review, we were interested in assessing a risk factor or exposure that increases the likelihood of developing a disease, in this case a musculoskeletal condition, rather than a prognostic factor that influences the outcome from or the course of a disease. Thus, we amended the ‘prognostic factor’ domain in QUIPS to reflect this. The following six domains were considered: study participation, study attrition, risk factor measurement, outcome measurement, study confounding, and statistical analysis and reporting. Each domain was assessed as having high, moderate or low risk of bias. Overall risk of bias was also assessed for each study; the designers of the QUIPS tool recommend that this is done by determining which domains (of the six) are most important and assigning low risk if a study is low in those domains [17]. In line with this recommendation, we categorised a study as having a ‘low risk of bias’ when the risk of bias was rated low on at least four of the six domains, and was rated low for both study attrition and study confounding. Two authors independently assessed each study (AW, SK). Consensus was used to resolve any disagreement and a third reviewer was consulted when required (CW).

### Data synthesis

We calculated pooled hazard ratios (HRs) of the effect of the exposure (musculoskeletal condition) on the outcome (chronic disease) and 95% confidence intervals (CIs) using the generic inverse variance method [18]. We used random effect models to incorporate heterogeneity between studies [19]. Not all studies reported HRs and 95% CI. Despite being modelled under different assumptions, incidence rate ratios are considered approximations of HRs and, therefore, were included in the meta-analysis [20]. Where the incidence rate and number of events were reported, these data were used to calculate the incidence rate ratio and standard error, respectively. Where the number of events was not reported, we attempted to contact authors for further data. Where authors did not respond to contact attempts, we estimated the standard error using the number of events derived from available data. The number of events was calculated using the number of patient years per group and the incidence rate per group. The patient years per group was calculated using the total number of people in each group (those with the musculoskeletal condition and those without), and the mean years of follow-up.

In the primary meta-analysis, we pooled estimates from all musculoskeletal conditions and chronic diseases. We pooled estimates using the most adjusted estimates from each study. Where possible, we also reported pooled estimates by musculoskeletal condition and by chronic disease. We undertook a secondary analysis using unadjusted estimates from each study.

Where there was more than one article reporting on the same patient sample, we included the data that was most clinically homogenous with the other included studies or included the more comprehensive exposure or outcome (i.e. all cancer rather than a specific cancer). If several estimates were reported from one study (e.g. men and women), where possible, we combined estimates using a fixed effects model to generate one estimate for the sample. When combining several estimates from within a study was not appropriate (i.e. where the unexposed group would be counted twice), we chose a single estimate based on clinical homogeneity with other studies in the meta-analysis, the less selective sample or interpretability of the clinical measures.

We used sensitivity analyses to test whether the primary adjusted meta-analysis was affected by overall risk of bias. This involved performing a meta-analysis including only studies at low risk of bias determined by the QUIPS tool, and comparing the pooled estimate with the primary analysis. We also used sensitivity analyses to assess whether the primary adjusted meta-analysis was affected by our decisions to choose between different exposures or outcomes reported within the one study. This involved performing meta-analyses whereby the alternative reported estimate was substituted in for our original choice, and the pooled estimates with the primary analysis were compared.

The impact of heterogeneity between studies was assessed using the I^2^ statistic with ≥ 50% considered substantial. Funnel plots to identify small-study effects were planned for analyses including at least 10 estimates [21]. All analyses were conducted using RevMan5 software [16].

## Results

### Study selection

**Fig. 1:**
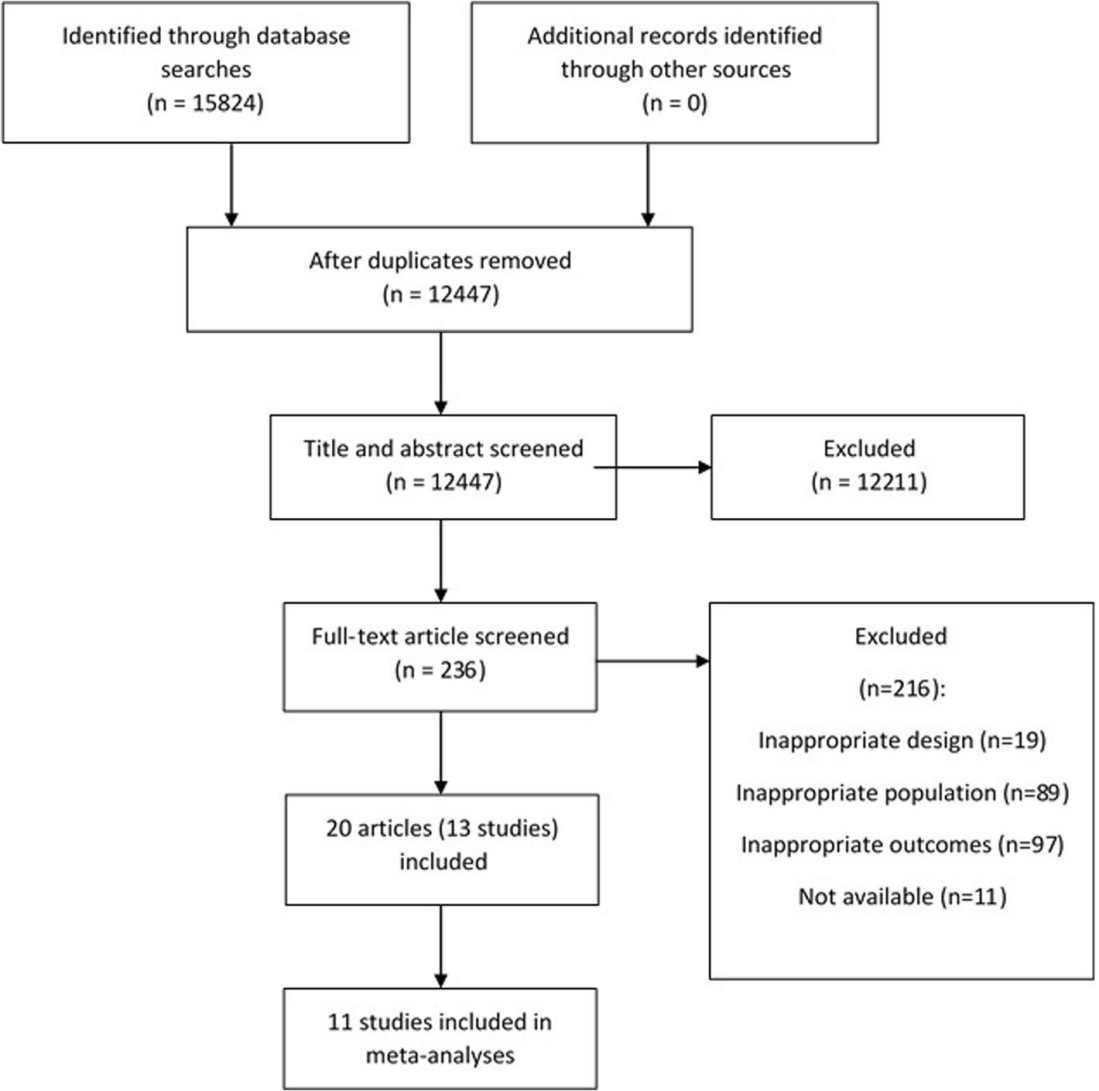
Flow diagram of study selection

The search identified 15,824 articles, of which 12,447 remained after removal of duplicates. There were 236 articles that remained after title and abstract screening; of these, 205 were excluded after assessment of the full text Additional file 2. Eleven abstracts were excluded as the full text or sufficient data were not available, confirmed by correspondence with authors or no response to contact attempts. Two abstracts with sufficient data and information to assess inclusion were included in the review. This left 20 articles [9, 12, 13, 22–38] that met the criteria for inclusion. The 20 articles reported on 13 studies, and there was sufficient data reported from 11 studies to be included in meta-analyses (Fig. 1).

### Study characteristics

The 13 studies included data from a total of 3,086,612 persons (mean follow-up 4–16 years) [9, 12, 13, 22, 24–26, 29, 31, 35–38]. Of those studies that reported a mean participant age, seven reported a mean of > 50 years [12, 13, 22, 24, 29, 31, 38], and three studies reported a mean of > 70 years [9, 35, 37]. Four studies were from Canada [13, 31, 35, 36], two from the United Kingdom [29, 38] and one each from the United States of America [25], Taiwan [22], the Netherlands [12], Italy [37], Spain [24], Australia [9] and Norway [26]. All studies were published in English. The musculoskeletal condition (exposure) was general OA in seven studies [13, 22, 25, 35–38], knee OA in three studies [12, 31, 37], hip OA in three studies [12, 31, 37], back pain in four studies [9, 24, 26, 29] and neck pain in one study [29]. The chronic disease (outcome) was cardiovascular disease in nine studies [9, 12, 13, 22, 25, 35–38], cancer in one study [29], diabetes in three studies [24, 31, 33] and obesity in one study [26]. All studies excluded participants who reported the outcome of interest at baseline. Descriptive data for all studies is provided in Additional file 1: Table S2.

### Risk of chronic disease from musculoskeletal conditions

**Fig. 2:**
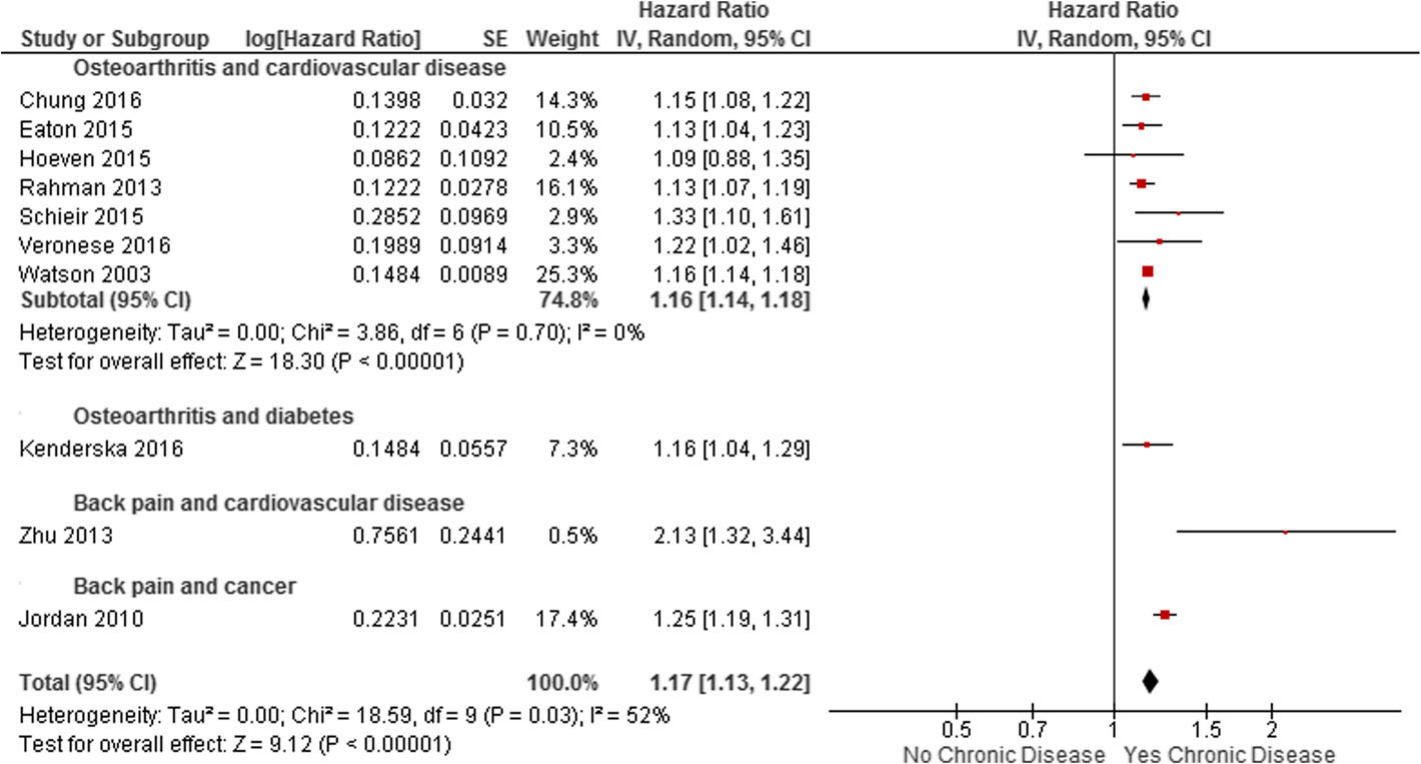
Meta-analysis of adjusted estimates of the association between musculoskeletal conditions and chronic disease

The 11 studies with sufficient data for meta-analysis reported 10 adjusted estimates from a total of 2,686,113 persons [9, 12, 13, 22, 25, 29, 31, 36–38] and five unadjusted estimates from a total of 612,873 persons [13, 22, 25, 35, 37]. The primary meta-analysis of adjusted estimates (Fig. 2) showed a statistically significant increased risk of chronic disease incidence from musculoskeletal conditions (HR 1.17, 95% CI 1.13–1.22, I^2^ 52%, 10 studies). Studies most often adjusted for age, sex, body mass index, hypertension, diabetes, hyperlipidaemia and smoking. A full list of adjustment variables per study are outlined in Additional file 1: Table S2. The unadjusted meta-analysis demonstrated a larger, statistically significant association (HR 1.39, 95% CI 1.23–1.58, I^2^ 94%, five studies).

### Analyses by condition

Combining all studies with adjusted estimates that assessed OA as the exposure revealed a statistically significant increased risk of chronic disease (HR 1.16, 95% CI 1.14–1.18, I^2^ 0%, eight studies). Seven of the eight studies that included OA as the exposure assessed the increased risk of cardiovascular disease [12, 13, 22, 25, 36–38]. Removal of the remaining study that assessed the increased risk of diabetes [31] did not change the results (Fig. 2). Combining the two studies with adjusted estimates that assessed OA as the exposure and diabetes as the outcome revealed a statistically significant increased risk of diabetes (HR 1.16, 95% CI 1.11–1.22, I^2^ 0%, two studies) [31, 33]. We were unable to perform analysis by back or neck pain due to the limited number of included studies of these conditions. Two individual studies of back pain found that those with back pain had an increased risk of cardiovascular disease [9] (HR 2.13, 95% CI 1.32–3.44) and cancer [29] (HR 1.25, 95% CI 1.19–1.32) compared to people without. The one study that assessed neck pain found that those with neck pain had an increased risk of cancer [29] (HR 1.20, 95% CI 1.09–1.31), compared to people without.

**Fig. 3:**
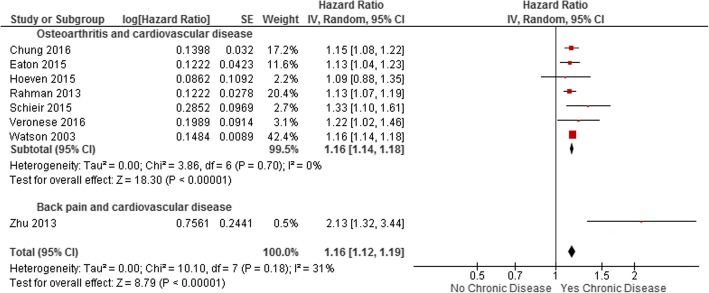
Meta-analysis of adjusted estimates of the association between musculoskeletal conditions and cardiovascular disease

Combining all musculoskeletal conditions as the exposure and cardiovascular disease as the outcome revealed a statistically significant increased risk of cardiovascular disease (HR 1.16, 95% CI 1.12–1.19, I^2^ 31%, eight studies) (Fig. 3). We were unable to perform analyses by other chronic disease (cancer, chronic respiratory disease, diabetes or obesity) due to the insufficient number of included studies of these conditions.

### Risk of bias assessment

Of the 13 included studies, 10% (*n* = 8) of the six domains showed a high risk of bias, 23% (*n* = 18) showed moderate risk, and 67% (*n* = 52) low risk. Risk of bias was highest in the ‘study confounding’ domain, with three studies at high risk [29, 35, 38] and four at moderate risk [9, 13, 24, 25]. Of the 10 studies included in the primary meta-analysis of adjusted estimates, three had an overall low risk of bias [12, 22, 31].

### Sensitivity analyses

Sensitivity analysis including the three low risk of bias studies demonstrated a statistically significant association between musculoskeletal conditions and chronic disease (HR 1.15, 95% CI 1.09–1.21, I^2^ 0%). These studies all assessed OA, two assessed cardiovascular disease [12, 22] and one diabetes [31].

We conducted sensitivity analyses of the primary adjusted meta-analysis, whereby we substituted in an alternative exposure (i.e. general OA vs. hip OA only vs. knee OA only) or outcome (i.e. cardiovascular disease vs. diabetes), from five studies [12, 13, 29, 31, 37] where multiple exposures or outcomes were reported. In all cases, using the alternative estimate did not alter the pooled HR by more than 0.2 and all estimates remained significant.

Substantial heterogeneity (I^2^ 52%) was present in the primary meta-analysis of adjusted estimates. Sensitivity analysis exploring this found that, when the two studies of back pain were removed and only studies of OA remained and heterogeneity dropped to 0%.

**Fig. 4:**
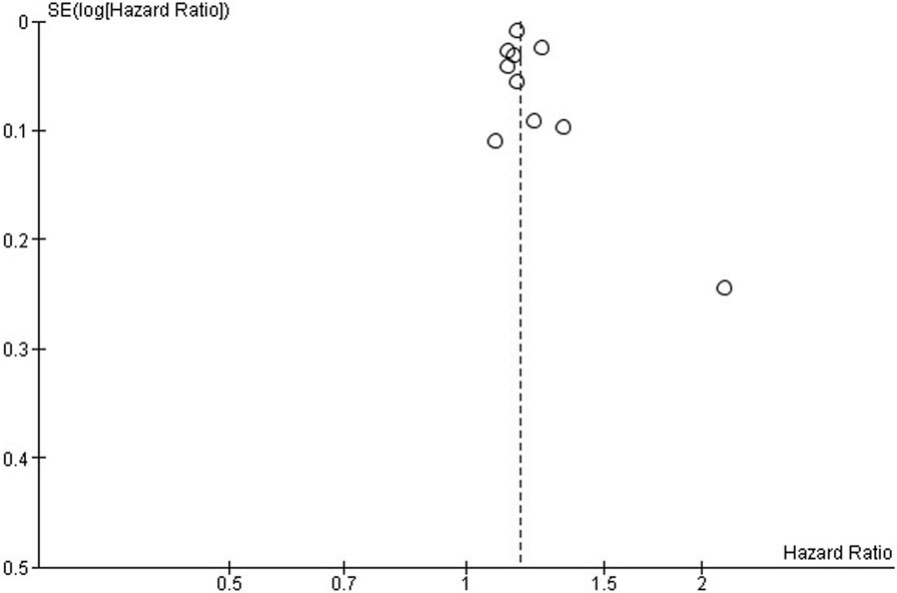
Funnel plot for adjusted estimates of the association between musculoskeletal conditions and chronic disease

Inspection of the funnel plot for the primary adjusted meta-analysis showed that the plot was symmetrical aside from one small-study outlier. Removal of the outlier [9] did not change the pooled estimate (Fig. 4).

## Discussion

This systematic review and meta-analysis, including data from 2,686,113 persons, showed that people with a musculoskeletal condition have a 17% increase in the risk of developing a chronic disease compared to people without such a condition. Most studies included OA as the exposure and cardiovascular disease as the outcome; analysis of these studies revealed that people who reported OA have a 16% increase in the risk of developing cardiovascular disease, compared to people without. Two individual studies concerning back pain and one of neck pain reported that those with back pain had an increased risk of cardiovascular disease and those with back or neck pain had an increased the risk of cancer. While our review question ultimately sought to assess a causal connection between common musculoskeletal conditions and chronic disease, we cannot draw strong conclusions due to poor adjustment, the analysis methods employed by the included studies, and a lack of studies investigating conditions other than OA and cardiovascular disease.

To the best of our knowledge, this is the first attempt at a meta-analysis of longitudinal cohort studies that estimates the risk of developing a chronic disease in people with highly prevalent and burdensome musculoskeletal conditions, neck or back pain, or OA of the knee or hip. This review was prospectively registered with the international prospective register of systematic reviews (PROSPERO) and has been reported following the MOOSE reporting guidelines. We used a comprehensive search strategy, studies were not limited by publication date or language, and we assessed risk of bias using a specific tool for observational studies. We reported analyses by condition, and conducted sensitivity analyses of studies that had low risk of bias and for studies that reported multiple exposures or outcomes, all of which provided precise estimates, very similar to that of the primary analysis.

The lack of sufficient low risk of bias studies to confidently test the full hypothesis represents a major limitation in the strength of the conclusions regarding the general hypothesis about musculoskeletal conditions. While there is more confidence that OA increases the risk of cardiovascular disease, the lack of studies assessing other conditions limits the generalisability of our results. Due to the small number of included studies we were unable to assess the effect of various study characteristics (e.g. age, sex, variation in the measurement of the exposure and outcome, etc.) on the observed estimates. Further, none of the studies in the review assessed latent exposure to musculoskeletal conditions. It is possible that participants who reported no musculoskeletal condition at baseline (i.e. unexposed), developed a musculoskeletal condition during follow-up (i.e. became exposed), but we could not assess this from the included studies. Furthermore, it is not clear if adjusting for this exposure would attenuate or amplify our estimate. Finally, while our assessment of funnel plots suggested that there was no evidence of small-study effects, we do not know the influence of the 11 studies for which a full text was not available.

Our intention was to synthesise studies that used methods enabling an assessment of causal effects. To do this, we restricted inclusion to longitudinal cohort studies to assess temporal relationships, and prioritised adjusted estimates over crude estimates to account for potential confounding. We did not find studies that satisfied all of Bradford Hill’s suggested criteria for casual inference (e.g. none estimated dose–response effects) nor did we find studies that used contemporary causal inference methods for observational data (e.g. a structured identification approach for selection of confounding variables [39, 40] or assessment of the effects of unmeasured or residual confounders [41–43]). As such, we are unable to infer a strong causal connection between musculoskeletal conditions and chronic diseases.

There is evidence to suggest that the relationships found in this review are biologically plausible, meaning that there are possible mechanisms by which musculoskeletal conditions may contribute to the development of chronic disease. For example, there is evidence to suggest that chronic inflammation from OA may increase the risk of cardiovascular disease [6]. Further, pain and disability from these conditions can often limit participation in physical activity and lead to higher weight gain, both of which are recognised risk factors for cardiovascular disease and cancer [6, 8, 9]. However, none of the included studies assessed possible causal mechanisms. While we did not intend to study mechanisms of the effect, our review provides useful evidence for one direction of accumulation of multimorbidity in people with chronic disease.

Our review focused on the most common and burdensome musculoskeletal conditions [1, 4], showing that they may play a role in the development of chronic disease. Most of the evidence to date focuses on OA. Given the high burden of back and neck pain, further research is required to examine the causal effects of these conditions on chronic diseases. Since it is impractical and unethical to randomise individuals to disease states (i.e. musculoskeletal conditions) better use of observational data is required. To facilitate assessment of causal effects, contemporary analysis methods that more accurately identify and account for confounders should be considered alongside observational data. These might include structural identification of confounders with directed acyclic graphs, matching on propensity scores, or the application of instrumental variable analysis to eliminate the effects of residual confounders [44]. In the context of policy and clinical practice, our findings suggest that considering musculoskeletal conditions in the prevention of chronic disease may be important. However, to inform this, it would be useful to formally identify the mechanisms by which musculoskeletal conditions could cause chronic disease. Understanding how musculoskeletal conditions interact with other co-existing risk factors could further inform targeted intervention strategies to reduce chronic diseases. Certainly, future trials that assess the feasibility and efficacy of targeting musculoskeletal conditions in chronic disease preventive strategies are warranted.

## Conclusions

This review found that musculoskeletal conditions may increase the risk of subsequent chronic disease. In particular, meta-analysis of over 2 million people shows that OA increases the risk of developing cardiovascular disease. The results suggest that prevention and early effective treatment of musculoskeletal conditions, such as OA and back and neck pain, may play a role in preventing other chronic diseases. Typical targets for chronic disease prevention currently include lifestyle risk factors such as poor diet, physical inactivity, alcohol consumption and smoking [45], but musculoskeletal conditions are currently largely ignored. Considering their high global burden, addressing musculoskeletal conditions via public health strategies may have an impact on other chronic diseases such as cardiovascular disease.

## Additional files

Additional file 1: **Table S1.** Search strategy. **Table S2.** Characteristics of included studies. (DOCX 61 kb)

Additional file 2: Excluded full texts and reasons for exclusion. (XLSX 34 kb)

## Abbreviations

CI: confidence interval
HR: hazard ratio
OA: osteoarthritis
QUIPS: Quality in Prognosis Studies
YLDs: years lived with disability
