# Supplementary material for: Musculoskeletal conditions may increase the risk of chronic disease: a systematic review and meta-analysis of cohort studies
Source: BMC Med. 2018 Sep 25;16:167. doi: 10.1186/s12916-018-1151-2 (PMC6154805; doi:10.1186/s12916-018-1151-2)
Supplement: Supplementary file 1 — Table S1. Search strategy. Table S2. Characteristics of included studies. (DOCX 61 kb) [file 12916_2018_1151_MOESM1_ESM.docx]

**Table S1.** Search strategy

| Database: MEDLINE  Search Strategy: | | |
| --- | --- | --- |
| **#** | **Searches** | **Results** |
| 1 | exp Overweight/ | 160682 |
| 2 | exp Obesity/ | 158483 |
| 3 | Obes*.tw. | 183304 |
| 4 | Body Mass Index/ | 92290 |
| 5 | Abdominal Fat/ | 1951 |
|  | **Combined at Set 39** |  |
| 6 | exp Osteoarthritis/ | 47474 |
| 7 | exp Back Pain/ | 31134 |
| 8 | Neck Pain/ | 5027 |
| 9 | (backache or neckache).tw. | 1965 |
| 10 | Musculoskeletal Pain/ | 1311 |
| 11 | Sciatica/ | 4441 |
| 12 | Neuralgia/ | 9508 |
| 13 | (dorsalgia or cervicalgia).tw. | 124 |
| 14 | ((Cervical Vertebrae or back or knee* or neck or spin* or hip* or lumb* or joint* or musculoske*) adj3 (pain* or ache* or aching or complaint* or stiff* or dysfunction* or disabil* or trauma* or disorder* or injur*)).tw. | 128911 |
| 15 | (osteoarthr* or osteo arthr*).tw. | 44030 |
| 16 | Coxarthr*.tw. | 1600 |
| 17 | 6 or 7 or 8 or 9 or 10 or 11 or 12 or 13 or 14 or 15 or 16 | 206206 |
| 18 | exp Cardiovascular Diseases/ | 2005418 |
| 19 | exp Cerebrovascular Disorders/ | 302083 |
| 20 | Cardiovascular.tw. | 286290 |
| 21 | Coronary.tw. | 304158 |
| 22 | Cerebrovascular.tw. | 38667 |
| 23 | (arteriosclero* or artherosclero*).tw. | 13919 |
|  | **Combined at set 40** |  |
| 24 | exp Neoplasms/ | 2790625 |
| 25 | exp Respiratory Tract Diseases/ | 1131784 |
| 26 | exp Diabetes Mellitus/ | 342867 |
| 27 | IDDM.tw. | 6752 |
| 28 | NIDDM.tw. | 6787 |
| 29 | MODY.tw. | 884 |
| 30 | glucose intoleran*.tw. | 7895 |
| 31 | (non insulin* depend* or noninsulin* depend* or non insulin?depend* or noninsulin?depend*).tw. | 11924 |
| 32 | ((typ* I or typ* II) adj6 diabet*).tw. | 14080 |
| 33 | (insulin* depend* or insulin?depend*).tw. | 28196 |
| 34 | exp Insulin Resistance/ | 62175 |
| 35 | (T1DM or T2DM).tw. | 8812 |
|  | **Combined at Set 41** |  |
| 36 | exp Cohort Studies/ | 1504401 |
| 37 | (cohort adj (analys* or stud*)).tw. | 100608 |
| 38 | 36 or 37 | 1523206 |
| 39 | 1 or 2 or 3 or 4 or 5 | 274880 |
| 40 | 18 or 19 or 20 or 21 or 22 or 23 | 2182009 |
| 41 | 26 or 27 or 28 or 29 or 30 or 31 or 32 or 33 or 34 or 35 | 395388 |
| **42** | **17 and 38 and (39 or 40 or 24 or 25 or 41)** | **5075** |

| Database: Embase Classic + Embase  Search Strategy: | | |
| --- | --- | --- |
| **#** | **Searches** | **Results** |
| 1 | Overweight.mp. | 66652 |
| 2 | exp obesity/ | 356737 |
| 3 | Obes*.tw. | 294232 |
| 4 | body mass/ | 230331 |
| 5 | abdominal fat/ | 3824 |
| 6 | 1 or 2 or 3 or 4 or 5 | 556494 |
| 7 | exp osteoarthritis/ | 99445 |
| 8 | exp backache/ | 79712 |
| 9 | neck pain/ | 15234 |
| 10 | (backache or neckache).tw. | 3043 |
| 11 | musculoskeletal pain/ | 6369 |
| 12 | sciatica/ | 780 |
| 13 | neuralgia/ | 8988 |
| 14 | (dorsalgia or cervicalgia).tw. | 254 |
| 15 | ((Cervical Vertebrae or back or knee* or neck or spin* or hip* or lumb* or joint* or musculoske*) adj3 (pain* or ache* or aching or complaint* or stiff* or dysfunction* or disabil* or trauma* or disorder* or injur*)).tw. | 196438 |
| 16 | (osteoarthr* or osteo arthr*).tw. | 69330 |
| 17 | Coxarthr*.tw. | 2692 |
| 18 | 7 or 8 or 9 or 10 or 11 or 12 or 13 or 14 or 15 or 16 or 17 | 344871 |
| 19 | exp cardiovascular disease/ | 3499892 |
| 20 | exp cerebrovascular disease/ | 454290 |
| 21 | Cardiovascular.tw. | 450368 |
| 22 | Coronary.tw. | 455986 |
| 23 | Cerebrovascular.tw. | 59187 |
| 24 | (arteriosclero* or artherosclero*).tw. | 25707 |
| 25 | 19 or 20 or 21 or 22 or 23 or 24 | 3703937 |
| 26 | exp neoplasm/ | 3848545 |
| 27 | exp respiratory tract disease/ | 2044455 |
| 28 | exp diabetes mellitus/ | 712474 |
| 29 | IDDM.tw. | 7586 |
| 30 | NIDDM.tw. | 7803 |
| 31 | MODY.tw. | 1462 |
| 32 | glucose intoleran*.tw. | 11690 |
| 33 | (non insulin* depend* or noninsulin* depend* or non insulin?depend* or noninsulin?depend*).tw. | 13818 |
| 34 | ((typ* I or typ* II) adj6 diabet*).tw. | 21103 |
| 35 | (insulin* depend* or insulin?depend*).tw. | 33156 |
| 36 | insulin resistance/ | 90992 |
| 37 | (T1DM or T2DM).tw. | 21464 |
| 38 | 28 or 29 or 30 or 31 or 32 or 33 or 34 or 35 or 36 or 37 | 766800 |
| 39 | cohort analysis/ | 220966 |
| 40 | (cohort adj (analys* or stud*)).tw. | 152667 |
| 41 | ((followup or follow up or longitudinal or prospective or retrospective) adj (analys* or stud*)).tw. | 524320 |
| 42 | 39 or 40 or 41 | 769164 |
| **43** | **18 and 42 and (6 or 25 or 26 or 27 or 38)** | **5328** |

| Database: PsycINFO  Search Strategy: | | |
| --- | --- | --- |
| **#** | **Searches** | **Results** |
| 1 | exp overweight/ | 19257 |
| 2 | obesity/ | 18315 |
| 3 | Obes*.tw. | 28742 |
| 4 | Body Mass Index/ | 3648 |
| 5 | Body Fat/ or Abdominal Fat.mp. | 1354 |
| 6 | 1 or 2 or 3 or 4 or 5 | 32471 |
| 7 | Back Pain/ | 3118 |
| 8 | (backache or neckache).tw. | 128 |
| 9 | exp Neuralgia/ | 795 |
| 10 | (dorsalgia or cervicalgia or Sciatica).tw. | 137 |
| 11 | ((Cervical Vertebrae or back or knee* or neck or spin* or hip* or lumb* or joint* or musculoske*) adj3 (pain* or ache* or aching or complaint* or stiff* or dysfunction* or disabil* or trauma* or disorder* or injur*)).tw. | 16756 |
| 12 | (osteoarthr* or osteo arthr*).tw. | 1418 |
| 13 | Coxarthr*.tw. | 9 |
| 14 | 7 or 8 or 9 or 10 or 11 or 12 or 13 | 18797 |
| 15 | exp cerebrovascular disorders/ | 21180 |
| 16 | exp Cardiovascular Disorders/ | 48654 |
| 17 | Cardiovascular.tw. | 22481 |
| 18 | Coronary.tw. | 8901 |
| 19 | Cerebrovascular.tw. | 5067 |
| 20 | (arteriosclero* or artherosclero*).tw. | 675 |
| 21 | 15 or 16 or 17 or 18 or 19 or 20 | 68454 |
| 22 | exp neoplasms/ | 39555 |
| 23 | exp respiratory tract disorders/ | 11830 |
| 24 | exp Diabetes Mellitus/ | 4332 |
| 25 | IDDM.tw. | 243 |
| 26 | NIDDM.tw. | 94 |
| 27 | MODY.tw. | 27 |
| 28 | glucose intoleran*.tw. | 270 |
| 29 | (non insulin* depend* or noninsulin* depend* or non insulin?depend* or noninsulin?depend*).tw. | 270 |
| 30 | ((typ* I or typ* II) adj6 diabet*).tw. | 906 |
| 31 | (insulin* depend* or insulin?depend*).tw. | 1032 |
| 32 | exp Resistance/ and exp Insulin/ | 173 |
| 33 | (T1DM or T2DM).tw. | 577 |
| 34 | 24 or 25 or 26 or 27 or 28 or 29 or 30 or 31 or 32 or 33 | 5914 |
| 35 | (cohort adj (analys* or stud*)).tw. | 13534 |
| 36 | ((followup or follow up or longitudinal or prospective or retrospective) adj (analys* or stud*)).tw. | 72436 |
| 37 | 35 or 36 | 84439 |
| **38** | **14 and 37 and (6 or 21 or 22 or 23 or 34)** | **77** |

| Database: MEDLINE In-Process & Other Non-Indexed Citations  Search Strategy: | | |
| --- | --- | --- |
| **#** | **Searches** | **Results** |
| 1 | Overweight.mp. | 5562 |
| 2 | obes*.mp. | 22159 |
| 3 | Body Mass Index.mp. | 13320 |
| 4 | Abdominal Fat.mp. | 434 |
| 5 | 1 or 2 or 3 or 4 | 31248 |
| 6 | (backache or neckache).tw. | 172 |
| 7 | (dorsalgia or cervicalgia or sciatica or Neuralgia).tw. | 1108 |
| 8 | ((Cervical Vertebrae or back or knee* or neck or spin* or hip* or lumb* or joint* or musculoske*) adj3 (pain* or ache* or aching or complaint* or stiff* or dysfunction* or disabil* or trauma* or disorder* or injur*)).tw. | 15365 |
| 9 | (osteoarthr* or osteo arthr*).tw. | 4558 |
| 10 | Coxarthr*.tw. | 37 |
| 11 | 6 or 7 or 8 or 9 or 10 | 20023 |
| 12 | Cardiovascular.tw. | 27608 |
| 13 | Coronary.tw. | 18829 |
| 14 | Cerebrovascular.tw. | 2839 |
| 15 | (arteriosclero* or artherosclero*).tw. | 414 |
| 16 | 12 or 13 or 14 or 15 | 44811 |
| 17 | (neoplasm* or cancer*).tw. | 114262 |
| 18 | (Respirat* or lung*).tw. | 54981 |
| 19 | Diabetes.tw. | 34841 |
| 20 | IDDM.tw. | 71 |
| 21 | NIDDM.tw. | 121 |
| 22 | MODY.tw. | 70 |
| 23 | glucose intoleran*.tw. | 567 |
| 24 | (non insulin* depend* or noninsulin* depend* or non insulin?depend* or noninsulin?depend*).tw. | 220 |
| 25 | ((typ* I or typ* II) adj6 diabet*).tw. | 927 |
| 26 | (insulin* depend* or insulin?depend*).tw. | 555 |
| 27 | (Insulin adj2 Resist*).tw. | 5658 |
| 28 | (T1DM or T2DM).tw. | 2372 |
| 29 | 19 or 20 or 21 or 22 or 23 or 24 or 25 or 26 or 27 or 28 | 38123 |
| 30 | (cohort adj (analys* or stud*)).tw. | 13478 |
| 31 | ((followup or follow up or longitudinal or prospective or retrospective) adj (analys* or stud*)).tw. | 32442 |
| 32 | 30 or 31 | 44858 |
| **33** | **11 and 32 and (5 or 16 or 17 or 18 or 29)** | **180** |

| Database: CINAHL  Search Strategy: | | |
| --- | --- | --- |
| **#** | **Query** | **Results** |
| **S43** | **S17 and S42 and (S6 or S24 or S25 or S26 or S37)** | **1,615** |
| S42 | S38 OR S39 OR S40 OR S41 | 335,884 |
| S41 | (MH "Retrospective Panel Studies") | 142 |
| S40 | (MH "Prospective Studies+") | 281,749 |
| S39 | TI ( ((followup or follow up or longitudinal or prospective or retrospective) n1 (analys* or stud*)) ) OR AB ( ((followup or follow up or longitudinal or prospective or retrospective) n1 (analys* or stud*)) ) | 99,701 |
| S38 | TI ( (cohort n1 (analys* or stud*)) ) OR AB ( (cohort n1 (analys* or stud*)) ) | 38,348 |
| S37 | S27 OR S28 OR S29 OR S30 OR S31 OR S32 OR S33 OR S34 OR S35 OR S36 | 123,146 |
| S36 | TI ( (T1DM or T2DM) ) OR AB ( (T1DM or T2DM) ) | 2,211 |
| S35 | (MH "Insulin Resistance+") | 18,367 |
| S34 | TI ( (insulin* depend* or insulin?depend*) ) OR AB ( (insulin* depend* or insulin?depend*) ) | 2,370 |
| S33 | TI ( ((typ* I or typ* II) n6 diabet*) ) OR AB ( ((typ* I or typ* II) n6 diabet*) ) | 1,632 |
| S32 | TI ( (non insulin* depend* or noninsulin* depend* or non insulin?depend* or noninsulin?depend*) ) OR AB ( (non insulin* depend* or noninsulin* depend* or non insulin?depend* or noninsulin?depend*) ) | 888 |
| S31 | (MH "Glucose Intolerance") | 2,295 |
| S30 | TI MODY OR AB MODY | 219 |
| S29 | TI NIDDM OR AB NIDDM | 607 |
| S28 | TI IDDM OR AB IDDM | 500 |
| S27 | (MH "Diabetes Mellitus+") | 109,105 |
| S26 | (MH "Respiratory Tract Diseases+") | 193,894 |
| S25 | (MH "Neoplasms+") | 343,517 |
| S24 | S18 OR S19 OR S20 OR S21 OR S22 OR S23 | 423,185 |
| S23 | TI ( (arteriosclero* or artherosclero*) ) OR AB ( (arteriosclero* or artherosclero*) ) | 475 |
| S22 | TI Cerebrovascular OR AB Cerebrovascular | 5,631 |
| S21 | TI Coronary OR AB Coronary | 48,182 |
| S20 | TI Cardiovascular OR AB Cardiovascular | 57,198 |
| S19 | (MH "Cerebrovascular Disorders+") | 70,895 |
| S18 | (MH "Cardiovascular Diseases+") | 388,522 |
| S17 | S7 OR S8 OR S9 OR S10 OR S11 OR S12 OR S13 OR S14 OR S15 OR S16 | 84,544 |
| S16 | TI Coxarthr* OR AB Coxarthr* | 37 |
| S15 | TI ( (osteoarthr* or osteo arthr*) ) OR AB ( (osteoarthr* or osteo arthr*) ) | 13,203 |
| S14 | TI ( ((Cervical Vertebrae or back or knee* or neck or spin* or hip* or lumb* or joint* or musculoske*) n3 (pain* or ache* or aching or complaint* or stiff* or dysfunction* or disabil* or trauma* or disorder* or injur*)) ) OR AB ( ((Cervical Vertebrae or back or knee* or neck or spin* or hip* or lumb* or joint* or musculoske*) n3 (pain* or ache* or aching or complaint* or stiff* or dysfunction* or disabil* or trauma* or disorder* or injur*)) ) | 54,485 |
| S13 | TI ( (dorsalgia or cervicalgia) ) OR AB ( (dorsalgia or cervicalgia) ) | 44 |
| S12 | (MH "Neuralgia") | 2,563 |
| S11 | (MH "Sciatica") | 1,176 |
| S10 | TI ( (backache or neckache) ) OR AB ( (backache or neckache) ) | 269 |
| S9 | (MH "Neck Pain") | 4,447 |
| S8 | (MH "Back Pain+") | 21,327 |
| S7 | (MH "Osteoarthritis+") | 18,222 |
| S6 | S1 OR S2 OR S3 OR S4 OR S5 | 112,360 |
| S5 | (MH "Abdominal Fat") | 1,088 |
| S4 | (MH "Body Mass Index") | 54,204 |
| S3 | TI Obes* OR AB Obes* | 48,636 |
| S2 | (MH "Obesity+") | 64,317 |
| S1 | "Overweight" | 14,236 |

| Database: SCOPUS Search Strategy: |
| --- |
| ALL((Osteoarthr* or "osteo arthr*" or backache* or neckache* or sciatica or neuralgia or dorsalgia or cervicalgia or ((Cervical Vertebrae or back or knee* or neck or spin* or hip* or lumb* or joint* or musculoske*) and (pain* or ache* or aching or complaint* or stiff* or dysfunction* or disabil* or trauma* or disorder* or injur*)) or coaxarthr*) AND (cohort or followup or follow up or longitudinal or prospective or retrospective) AND (Overweight or obes* or "Body mass index" or "abdominal fat" or cardiovascular or cerebrovascular or coronary or arteriosclero* or artherosclero* or neoplasm* or cancer* or respirat* or lung* or diabet* or iddm or niddm or mody or "glucose intoleran*" or insulin* or noninsulin or "type 1" or "type 2" of t1dm or t2dm)) |

| Database: WEB OF SCIENCE Search Strategy: |
| --- |
| TITLE: ((Osteoarthr* or "osteo arthr*" or backache* or neckache* or sciatica or neuralgia or dorsalgia or cervicalgia or ((“Cervical Vertebrae” or back or knee* or neck or spin* or hip* or lumb* or joint* or musculoske*) and (pain* or ache* or aching or complaint* or stiff* or dysfunction* or disabil* or trauma* or disorder* or injur*)) or coaxarthr*)) AND TITLE: ((cohort or followup or “follow up” or longitudinal or prospective or retrospective))AND TITLE: ((Overweight or obes* or "Body mass index" or "abdominal fat" or cardiovascular or cerebrovascular or coronary or arteriosclero* or artherosclero* or neoplasm* or cancer* or respiratory or lung* or diabet* or iddm or niddm or mody or "glucose intoleran*" or insulin* or noninsulin or "type 1" or "type 2" of t1dm or t2dm)) |

| **Table S2.** Characteristics of included studies | | | | | | |  |  |  |
| --- | --- | --- | --- | --- | --- | --- | --- | --- | --- |
| **Source**  **(Country)** | **Population Description** | **Patients with MSK, No./Total No. (%)^♯^** | **Age, y^♯^** | **Men, No. (%)^♯^** | **Measure of MSK** | **Measure of Chronic Disease** | | **Follow-up time, y** | **Adjustment variables** |
| Chung et al, 2016  (Taiwan)  (22, 23) | National Health Insurance Research Database (covers 99% of residents & 96% of healthcare institutions Taiwan) | 46,042/92,084 (50) | Mean (SD) 60.6 (14.1) | 37740 (41) | OA (OA ICD9 codes at more than 3 healthcare visits) | CVD (ACS diagnosis, ICD9 codes) | | Mean OA 8.0 ± 1.5  Mean Control  7.9 ± 1.7 | Age, sex, comorbidities (HTN, DM, hyperlipidaemia, stroke and congestive heart failure) |
| Dario et al, 2017*  (Spain)  (24) | Adult twins from the Murcia Twin Registry, born 1940-1966 in the Murcia region | 675/2096 (32) | Mean (SD) 53.6 (7.3)† | 940 (45)† | LBP (self-reported chronic LBP from the Spanish National Health Survey, LBP persisting for ≥6 months including seasonal or recurrent episodes) | Diabetes (self-reported diabetes from the Spanish National Health Survey confirmed by diagnosis by physician recorded in healthcare records) | | Mean NR  Total follow-up 2-4 | Age, sex, BMI, smoking, physical activity |
| Eaton et al, 2015  (USA)  (25) | Postmenopausal women; Women’s Health Initiative | 40,421/96,047 (42) | NR | 0 (0) | OA (self-reported OA & self-reported joint pain) | CVD (MI and CHD mortality, medical records & death certificate) | | NR | Age, race, SES (income, education), CHD risk factors (DM, hyperlipidemia, HTN, smoking, family history of CHD, BMI), Lifestyle risk factors (physical acitivity, total calories/day, alternative healthy eating index, alcohol), medications (aspirin, NSAIDs, beta-blockers, statins), access to care (personal physician, insurance, modified Charlson co-morbidity index), psychosocial risk factors (marital status, social support, social strain, depression) |
| Heuch et al, 2013*  (Norway)  (26) | Residents 30-69yrs in county of Nord-Trondelag (HUNT 2 & HUNT 3) | 6568/25450 (26) | Range  30-69 | 11402 (45) | LBP (self-reported chronic LBP, LBP persisting for ≥3 months during the past yr) | Obesity (BMI divided into 3 groups: <25, 25-29.9, 30+) | | Mean NR  Total follow-up 11 | Age, education, work status, physical activity at work & in leisure time, smoking, blood pressure, lipid levels, time between last meal & blood sampling, BMI at baseline. |
| Hoeven et al, 2015  (Netherlands)  (12, 27-28) | Residents 55yrs+ in Ommoord district, Rotterdam for at least 1yr | 336/4648 (7) | Mean 67.6 ± 7.9 | 1813 (39) | Knee OA, Hip OA (radiographic [K&L score ≥2] & joint complaints in last month) | CVD (Total CVD: MI, surgical or percutaneous revascularisation, coronary mortality, stroke [ischaemic & haemorrhagic], GP medical records confirmed by patient’s physician) | | Median 14.4 | Age, sex, BMI, DM, HTN, total chol. HDL chol. Ratio, smoking |
| Jordan et al,  2010  (UK)  (29,30) | Persons 50yrs+ from the General Practice Research Database (covering ~5% of the UK population) | 9259/49513 (19) | Mean (SD) Back 65.0 (10.9)  Mean (SD) controls 66.5 (10.8) | Back 4223 (46)  Controls 18145 (45) | Back pain, neck pain (at least 1 consult for back or neck pain, Read or Oxmis Code) | Cancer (consult for malignant or pre-malignant neoplasm, Read or Oxmis Code) | | Median Back 9.7  Median  Controls 9.4 | Age & sex standardised |
| Kendzerska et al,  2016  (Canada)  (31, 32) | Cohort of residents 55yrs+ | 2431/16362 (15) | Median 68‡ | 6381 (39)‡ | Knee OA, Hip OA (self-reported symptomatic OA, swelling, pain, or stiffness in any joint lasting ≥6 weeks in the past 3 months, and indication on a joint homunculus that a knee or hip was ‘troublesome’) | Diabetes (diagnosis as defined in health administration data) | | Median 13 | Age, sex, BMI, income, pre-existing comorbidities (CVD, HTN), prior primary care exposure |
| Rahman et al, 2013  (Canada)  (13, 33,34) | Random representative sample of all individuals 20yrs+ in the MSP or British Columbia | 12745/49631 (26) | Mean OA 58.2 ± 14.5  Mean controls 57.5 ± 14.3 | OA patients 5098 (40)  Controls 15123 (41) | OA (diagnosis by health professional ICD9/10 codes) | CVD, Diabetes (hospital discharge records ICD9/10 codes) | | Mean 13 | History of DM HTN, hyperlipidemia, COPD, Charlson score, BMI, and SES |
| Ray et al, 2005  (Canada)  (35) | Provincial health care administrative databases of 1.5 million senior residents 65yrs+ of Ontario | 172953/372953 (46) | Mean (SD) OA 74.9 (6.8)  Mean controls (SD) 74.7 (7.0) | OA 67429 (39)  Controls 82574 (41) | OA (hospital records ICD9 codes) | CVD (diagnosis or surgical treatment of coronary artery disease, stroke, PAD or aneurysm or dissection of the aorta, healthcare database records ICD9 codes) | | Mean NR  Total follow-up 7 | Unadjusted analyses only |
| Schieir et al, 2015  (Canada)  (36) | Participants 18yrs+, National Population Health Survey | NR/12591 (NR) | Mean 43.0 (0.2) | 5728 (45) | Arthritis (self-reported arthritis excluding RA and fibromyalgia) | CVD (Heart disease, self-reported health professional diagnosis, or heart disease death ICD10 codes, cause of death confirmed against death database) | | Mean NR  Total follow-up 16 | Age, education, high blood pressure, DM, BMI, smoking, physical activity, other chronic conditions and use of pain relievers |
| Veronese et al, 2016  (Italy)  (37) | Participants 65yrs+ from the Progetto Veneto Anziano cohort study | 1336/2158 (62) | Mean 75.4 ± 7.6 | 805 (37) | OA, Knee OA, Hip OA (medical history, clinical records, previous radiographic reports, OA-related pain, and examination of movement) | CVD (CAD, stroke, TIA, heart failure, PAD, CVD-related hospitalisation, CVD-related death, physical examination, medical history, ICD9 codes) | | Mean 4.4 ± 1.2 | Age, sex, waist-to-hip ratio, education level, baseline COPD, atrial fibrillation, HTN, DM, baseline low-dose aspirin, antihypertensives, and NSAIDs, number of medications, smoking, ADLs, Mini-Mental State Exam, Geriatric Depression scale score, glycosylated hemoglobin levels, total cholesterol, serum uric acid, estimated GFR and erythrocyte sedimentation rate, ankle brachial index, Short Physical Performance Battery, hand grip strength |
| Watson et al, 2003  (UK)  (38) | Persons 40yrs+ from the General Practice Research Database (covering ~6% of the UK population) | 163274/2361918 (7) | Mean (SD) men 54.5 (13.7)‖  Mean (SD) women 57.2 (15.1)‖ | 1106064 (47) | OA (patient record for diagnosis of OA) | CVD (All vascular events, patient records of fatal or nonfatal MI or cerebrovascular event, or sudden/unexplained death) | | Mean men 4.7  Mean women 4.8 | Age & sex standardised |
| Zhu et al, 2013  (Australia)  (9) | Participants 70-85yrs from the Calcium Intake Fracture Outcome Random selection of women 70yrs+ on the western Australia electoral roll | 323/1161 (29) | Mean Daily back pain 75.1 ± 2.7  Mean Infrequent back pain 75.3 ± 2.7 | 0 (0) | Back pain (self-reported daily back pain) | CVD (CHD: Ischemic heart disease and angina ICD10 codes, self-report patient diary with healthcare professional assistance & hospital morbidity data system & primary care physician records) | | Mean NR  Total follow-up 5 | Baseline age, BMI, smoking history, analgesia use, DM, CVD, hypercholesterolemia & HTN |
| *Not included in the meta-analyses  **^♯^** Data is presented on the exposure included in the primary adjusted meta-analysis unless otherwise indicated  † For whole sample including participants with Chronic LBP, Neck pain, Spinal pain and No LBP, Neck pain or Spinal pain  ‡ For whole sample including participants with Knee OA, Hip OA and No OA  ‖ For whole sample including participants with Osteoarthritis, Rheumatoid Arthritis and No arthritis  Abbreviations: SD= standard deviation, OA= osteoarthritis, ICD= International Classification of Diseases codes, CVD=cardiovascular disease, ACS= acute coronary syndrome, HTN= hypertension, DM= diabetes, LBP= low back pain, NR= not reported, MI- myocardial infarction, CHD= coronary heart disease, SES= socioeconomic status, BMI= body mass index, NSAIDs=nonsteroidal anti-inflammatory drugs, yrs= years, K&L score= Kellgren and Lawrence scale, GP=general practitioner, chol.=cholesterol, HDL= high density lipoprotein, MSP=Medical Services Plan, COPD= chronic obstructive pulmonary disease, PAD= peripheral artery disease, RA= rheumatoid arthritis, CAD=coronary artery disease, TIA= transient ischemic attack, ADLs= activities of daily living, GFR= glomerular filtration rate. | | | | | | | | | |
